# Supplementary material for: Resolving the full spectrum of human genome variation using Linked-Reads
Source: Genome Res. 2019 Apr;29(4):635–45. doi: 10.1101/gr.234443.118 (PMC6442396; doi:10.1101/gr.234443.118)
Supplement: Supplemental Material [file supp_gr.234443.118_Supplemental_longranger-2.2.zip › longranger-2.2.tar.bz2!/lr2.2-public/lib/stan/cmdstan/stan/doc/api/html/fwd_2functions_2fmod_8hpp_source.html]

Stan: src/stan/agrad/fwd/functions/fmod.hpp Source File


|  |  |
| --- | --- |
|  | Stan  2.5.0  probability, sampling & optimization |


- Main Page
- Related Pages
- Namespaces
- Classes
- Files

- File List
- File Members

All Classes Namespaces Files Functions Variables Typedefs Enumerations Enumerator Friends Macros Pages

- src
- stan
- agrad
- fwd
- functions

fmod.hpp

Go to the documentation of this file.

1 #ifndef STAN\_\_AGRAD\_\_FWD\_\_FUNCTIONS\_\_FMOD\_HPP

2 #define STAN\_\_AGRAD\_\_FWD\_\_FUNCTIONS\_\_FMOD\_HPP

3

4 #include <stan/agrad/fwd/fvar.hpp>

5 #include <stan/meta/traits.hpp>

6 #include <stan/math/functions/value\_of.hpp>

7 #include <stan/math/constants.hpp>

8

9 namespace stan {

10

11  namespace agrad {

12

13  template <typename T>

14  inline

15  fvar<T>

16  fmod(const fvar<T>& x1, const fvar<T>& x2) {

17  using std::fmod;

18  using std::floor;

19  return fvar<T>(fmod(x1.val\_, x2.val\_),

20  x1.d\_ - x2.d\_ \* floor(x1.val\_ / x2.val\_));

21  }

22

23  template <typename T>

24  inline

25  fvar<T>

26  fmod(const fvar<T>& x1, const double x2) {

27  using std::fmod;

28  using stan::math::value\_of;

29  if (unlikely(boost::math::isnan(value\_of(x1.val\_))

30  || boost::math::isnan(x2)))

31  return fvar<T>(fmod(x1.val\_,x2),stan::math::NOT\_A\_NUMBER);

32  else

33  return fvar<T>(fmod(x1.val\_, x2), x1.d\_ / x2);

34  }

35

36  template <typename T>

37  inline

38  fvar<T>

39  fmod(const double x1, const fvar<T>& x2) {

40  using std::fmod;

41  using std::floor;

42  return fvar<T>(fmod(x1, x2.val\_), -x2.d\_ \* floor(x1 / x2.val\_));

43  }

44  }

45 }

46 #endif

boost::math::isnan

bool isnan(const stan::agrad::var &v)

Checks if the given number is NaN.

**Definition:** boost\_fpclassify.hpp:69

stan::math::NOT\_A\_NUMBER

const double NOT\_A\_NUMBER

(Quiet) not-a-number value.

**Definition:** constants.hpp:53

stan::agrad::fvar

**Definition:** fvar.hpp:13

stan::agrad::floor

fvar< T > floor(const fvar< T > &x)

**Definition:** floor.hpp:15

stan::agrad::fmod

var fmod(const double a, const var &b)

Return the floating point remainder after dividing the first scalar by the second variable (cmath)...

**Definition:** fmod.hpp:139

stan::agrad::value\_of

T value\_of(const fvar< T > &v)

Return the value of the specified variable.

**Definition:** value\_of.hpp:16

traits.hpp

stan::math::value\_of

double value\_of(const T x)

Return the value of the specified scalar argument converted to a double value.

**Definition:** value\_of.hpp:24

unlikely

#define unlikely(x)

**Definition:** likely.hpp:9

fvar.hpp

stan::agrad::fmod

fvar< T > fmod(const fvar< T > &x1, const fvar< T > &x2)

**Definition:** fmod.hpp:16

value\_of.hpp

stan::agrad::fvar::d\_

T d\_

**Definition:** fvar.hpp:16

constants.hpp

stan::agrad::fvar::val\_

T val\_

**Definition:** fvar.hpp:15


---

[ Stan Home Page ]

*© 2011–2014,
Stan Development Team.*
